# Transcriptome analysis reveals the regulatory mechanism by which *MdWOX11* suppresses adventitious shoot formation in apple

**DOI:** 10.1093/hr/uhac080

**Published:** 2022-04-11

**Authors:** Jiangping Mao, Doudou Ma, Chundong Niu, Xiaolong Ma, Ke Li, Muhammad Mobeen Tahir, Shiyue Chen, Xiuxiu Liu, Dong Zhang

## Abstract

Adventitious shoot (AS) regeneration accelerates plant reproduction and genetic transformation. *WOX11* is involved in many biological processes, but its regulation of AS regeneration has not been reported. Here, we showed that the genotype and CK/IAA ratio of apple leaves were the key factors that affected their capacity for AS formation. Moreover, the expression level of *MdWOX11* was negatively correlated with the capacity for AS formation. Phenotypic analysis of *MdWOX11* transgenic plants showed that overexpression of *MdWOX11* inhibited AS formation. Endogenous hormone analysis demonstrated that the contents of auxin (IAA), cytokinin (CK), and abscisic acid (ABA) were higher in *MdWOX11-RNAi* plants than in *MdWOX11-OE* transgenic plants*.* We used RNA sequencing to examine the transcriptional responses of genes in *MdWOX11-RNAi* and *MdWOX11-OE* transgenic apple plants at different AS stages. We identified 8066 differentially expressed genes and focused our analysis on those involved in the IAA, CK, ABA, and gibberellin (GA) hormone signaling pathways. The expression of genes related to the CK signaling pathway and shoot development was higher in GL-3 than in *MdWOX11-OE* transgenic plants during the callus and AS emergence stages. However, the expression of *MdCKX5* was higher in *MdWOX11-OE* transgenic plants than in GL3 and *MdWOX11-RNAi* transgenic plants. Yeast one-hybrid (Y1H) assays, dual-luciferase reporter assays, and ChIP-qPCR showed that MdWOX11 binds to the promoter of *MdCKX5*, and a dual-luciferase reporter assay showed that MdWOX11 enhanced the promoter activity of *MdCKX5.* We concluded that *MdCKX5* acts downstream of *MdWOX11* to control AS formation, and we built a regulatory model of the suppression of AS formation by *MdWOX11* in apple.

## Introduction

Plant regeneration refers to the process by which cells, tissues, or structures that have been damaged or partly subjected to environmental stress respond by self-repair or structural replacement [1]. The acquisition of regeneration ability depends on the totipotency or pluripotency of plant cells and helps to ensure the developmental plasticity of the plant. Adventitious shoot (AS) regeneration is not only an effective approach for rapid plant propagation but also provides an experimental system for studies of plant development and genetic transformation. The AS developmental process has four main stages: the acquisition of cell pluripotency, the formation of bud primary meristematic tissue, the establishment of restricted bud primordium cells, and the germination and elongation of the bud [2]. To date, the capacity to regenerate an entire plant from AS organs through *in vitro* culture has been reported in various plants, including *Arabidopsis* [3], tomato [4], poplar [5], and others. Dependable processes for AS formation have been developed for model plants, but methods of improving AS formation in apple remain to be studied.

Apple (*Malus domestica*) is a major commercial fruit tree cultivated worldwide. Apple genetic transformation provides a new approach for apple breeding; the breeding cycle can be shortened and breeding efficiency improved by combining genetic transformation technology with conventional breeding. The leaf disc method is used for apple genetic transformation, meaning that regenerated buds must be obtained from the leaf. Therefore, establishing a complete and efficient *in vitro* leaf regeneration system is a prerequisite for successful genetic transformation. AS regeneration in apples is affected by multiple factors, including genotype, explant maturity, medium, hormone concentration, culture conditions, and so on. Genotype is the decisive factor for the regeneration of explants, and genotypes differ significantly in regeneration efficiency under identical culture conditions [6]. Nonetheless, the AS regeneration ability of apple leaves from different genotypes requires further clarification.

It is well known that a high ratio of auxin (IAA) to cytokinin (CK) promotes root regeneration, whereas a high ratio of CK to IAA promotes bud regeneration [7]. In general, AS formation proceeds in two sequential steps: first, callus formation is promoted by culture on callus induction medium (CIM) with a high IAA level, and then calli are transferred to shoot induction medium (SIM) with a high CK level to produce AS. IAA and CK are the two most important hormones that influence AS formation [8]. Studies have found that the size of the shoot apical meristem (SAM) and the formation of primordial organ units are negatively affected in CK-deficient plants, providing direct evidence that CK enhances SAM activity [9]. Exogenous gibberellin (GA) has been found to reduce the formation of buds, whereas the GA biosynthesis inhibitor paclobutrazol can improve the bud regeneration rate [10]. In addition, abscisic acid (ABA) promotes shoot regeneration in *Arabidopsis* [11]. Nonetheless, the role of these hormones in the AS regeneration of apples has not been widely studied.

Some studies have shown that bud regeneration depends on the induction of *WUS* and *CLV3* expression in the callus [12]. In addition, *WUSCHEL-RELATED HOMEOBOX GENE 5* (*WOX5*) expression is inhibited during bud regeneration [13]. *WUS*, *WOX5*, and *WOX11* all belong to the *WOX* family; whether *WOX11* regulates AS regeneration has not been reported. Most research on *WOX11* has focused on the regulation of adventitious root formation. For example, *WOX11* promotes crown root development and *de novo* root organogenesis in rice and *Arabidopsis* [14, 15]; it also participates in crown shoot emergence and development [16]. The *WOX11–LBD16* pathway promotes pluripotency acquisition in *Arabidopsis* callus cells [17]. However, the regulatory mechanism by which *WOX11* influences AS development in woody plants is poorly understood.

Hormone-related genes are also involved in AS formation. Some studies have demonstrated that IAA-related genes such as auxin response factors (*ARFs*), *YUCCA*, and *GH3* participate in AS regeneration. *ARF5* may influence callus bud formation through the transcriptional activation of *STM* and *CRF2* [18]. Transgenic plants overexpressing *ARF10* showed a higher level of bud regeneration than wild-type explants, and this was accompanied by high expression levels of *CLV3*, *CUCs*, and *WUS* [19, 20]. High expression levels of the IAA synthesis-related gene *YUCCA* promote AS regeneration ability [21], and this ability was significantly inhibited in the *pin1* mutant [12]. In apple, the overexpression of *MsGH3.5* inhibits shoot development through the IAA and CK pathways [22]. Many components of CK biosynthesis and signal transduction pathways are considered to be key drivers of AS regeneration. *AHK2*, *AHK3*, and *AHK4* encode CK-receptor histidine kinases that play an active role in AS generation. Deletion of *AHK2*, *AHK3*, and *AHK4* genes in mutants caused a decrease in AS regeneration ability, even when the mutants were induced on a medium with high CK levels [23, 24]. The CK oxidase gene *CKX5* is expressed in the meristem [25], and *CKX1-*overexpressing plants exhibit slow shoot growth and lower CK content [26]. *A-ARR* genes negatively regulate the CK signaling pathway, and AS regeneration ability is improved in an *A-ARR* mutant [27]. The *B-ARR* genes play a positive regulatory role in the CK pathway [28, 29], and *B-ARR* genes regulate *WUS* expression during shoot regeneration in *Arabidopsis* [30]. How CK-related genes regulate AS regeneration in apples remains to be studied.

At present, the mechanism by which *MdWOX11* regulates AS formation in apples is not well characterized. In the present study, we show that there are differences in the capacity for AS formation among apple leaves of different genotypes and that an appropriate CK/IAA ratio promotes AS formation. We further demonstrate that overexpression of *MdWOX11* inhibits the emergence and development of AS, and we use RNA sequencing (RNA-seq) to document changes in gene expression associated with the overexpression and suppression of *MdWOX11*. Finally, we show that *MdCKX5* acts downstream of *MdWOX11* to control AS formation. This study sheds light on the mechanism by which *MdWOX11* regulates AS formation and provides insight into the overall regulation of AS formation in apple rootstocks.

## Results

### AS regeneration in different apple rootstocks

**Figure 1:**
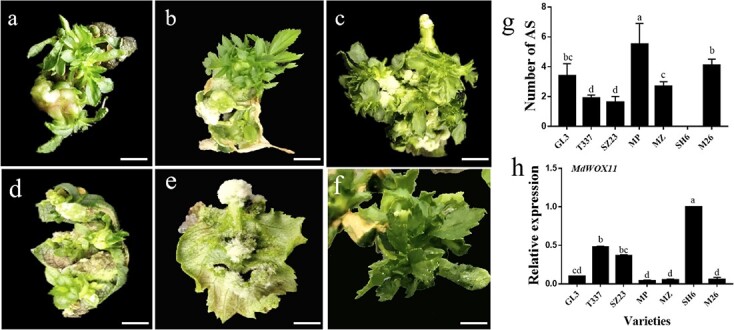
Morphological observations of the regeneration of adventitious shoots (AS) in six apple rootstocks. The hormone combination was TDZ 2 mg·L^−1^ and NAA 0.5 mg·L^−1^. a: T337; b: SZ23; c: *Malus prunifolia* (MP); d: *Malus zumi* (MZ); e: SH6; f: M26. Bar = 0.5 cm. g: the number of AS after 10 weeks of culture on leaves of six apple rootstocks and GL-3. h: Analysis of *MdWOX11* expression in leaves of six apple rootstocks and GL-3. Values represent the mean ± SE of three biological replicates; letters indicate significant differences between means (*P* < 0.05).

To analyze the effect of genotype on AS formation, fully-opened leaves were selected from terminal microshoots of different apple rootstocks. Results showed that AS regeneration ability differed significantly among apple leaves of different rootstocks when a hormone combination of 2 mg·L^−1^ thidiazuron (TDZ) and 0.5 mg·L^−1^ 1-naphthaleneacetic acid (NAA) was used. On average, 3.5 AS were produced from each leaf of *Malus prunifolia* (MP) stem cuttings, whereas 4.1 were produced from M26 leaves and 1.6 from SZ23 leaves. Under the same culture conditions, the AS regeneration number of MP was much higher than that of SZ23, but the regeneration ability of SZ23 was significantly higher than that of SH6. Overall, the AS regeneration abilities of the rootstocks were ranked as follows: M26 > MP > *Malus zumi* > T337 > SZ23 > SH6 (Figure 1a–g). We next analyzed the expression of *MdWOX11* in these apple rootstocks when the leaves were cultured on regenerative medium and found that it was highest in SH6 and lowest in MP, which had high AS regeneration ability. The expression of *MdWOX11* was therefore negatively correlated with AS regeneration ability (Figure 1h).

### AS regeneration in GL-3 and *MdWOX11* transgenic plants

**Figure 2:**
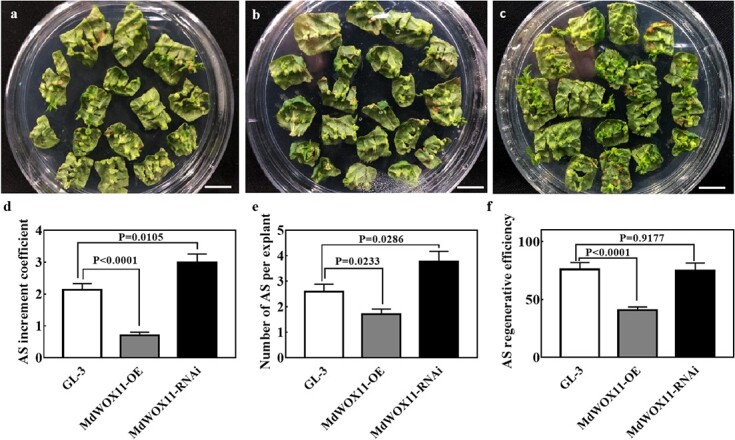
Regeneration of adventitious shoots (AS) in GL-3 and the *MdWOX11* transgenic plants after 10 weeks of culture. The phenotype of AS in a: GL-3; b: *MdWOX11-OE*; and c: *MdWOX11-RNAi*. Bar = 1 cm. d: AS increment coefficient; values represent the mean ± SE (n = 108). e: Number of AS per explant; values represent the mean ± SE (n = 9). f: AS regenerative efficiency; values represent the mean ± SE (n = 9). Significant differences were determined using the Student’s *t* test.

To analyze the function of *MdWOX11* during AS formation, we obtained *MdWOX11-OE* and *MdWOX11-RNAi* transgenic plants by an *Agrobacterium*-mediated method. *MdWOX11* expression in three OE and three RNAi lines is shown in Figure S1; interference with *MdWOX11* inhibited the expression of *MdWOX11* but not of *MdWOX3*, *MdWOX8*, and *MdWOX9*. *MdWOX11OE-23#* and *MdWOX11RNAi-10#* were selected for AS induction. The expression of *MdWOX11* was higher in *MdWOX11-OE* transgenic plants than in GL-3 and *MdWOX11-RNAi* transgenic plants during AS formation (Figure S1). The AS regeneration phenotypes of GL-3 and the *MdWOX11* transgenic lines are shown in Figure 2. When leaves from stem cuttings were cultured on AS induction medium for 10 weeks, GL-3 and *MdWOX11* transgenic plants differed in their AS regeneration ability. The average number of regenerated AS per leaf and the AS increment coefficient for *MdWOX11-RNAi* transgenic plants were clearly higher than those for GL-3. However, no significant difference in AS regenerative efficiency was noted between them. By contrast, the AS regeneration ability of *MdWOX11-OE* transgenic lines was significantly lower than that of GL-3 and *MdWOX11-RNAi* plants. The average number of regenerated AS in *MdWOX11-OE* transgenic plants was lower than that in GL-3 and *MdWOX11-RNAi* plants. The AS regeneration frequency and AS increment coefficient were also significantly lower in *MdWOX11-OE*. These results indicated that *MdWOX11* inhibited AS formation.

**Figure 3:**
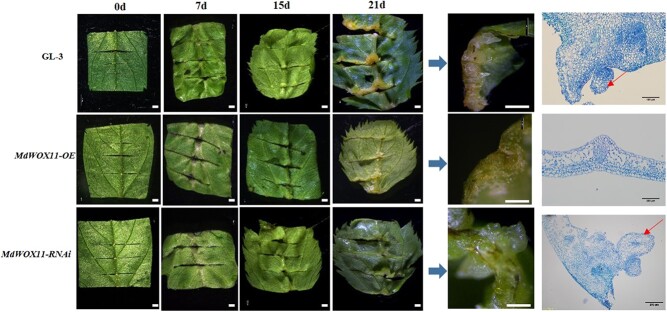
Phenotypic and anatomical observations during the formation of adventitious shoots (AS) in the leaves of GL-3 and *MdWOX11* transgenic plants (*MdWOX11-OE* and *MdWOX11-RNAi*) at 0, 7, 15, and 21 d. In the photographs, bar = 0.1 cm. In micrographs, the red arrow indicates the AS primordium.

We next observed the anatomical and morphological features of AS formation in the leaves of wild-type GL-3 and *MdWOX11* transgenic plants. The leaves were significantly enlarged after 3–7 days of AS-inducing culture, and a small amount of callus was visible. The callus became larger at approximately 2 weeks. At 21 d, most GL-3 and *MdWOX11-RNAi* transgenic leaves showed obvious white AS, but *MdWOX11-OE* leaves did not show AS formation at 21 d (Figure 3). Based on these initial observations, we chose to sample leaves for hormone content measurements and RNA-seq at three specific times of AS development: 0 d (T1), 15 d (T2), and 21 d (T3).

### Hormone levels and hormone ratios during AS formation in GL-3 and *MdWOX11* transgenic plants

**Figure 4:**
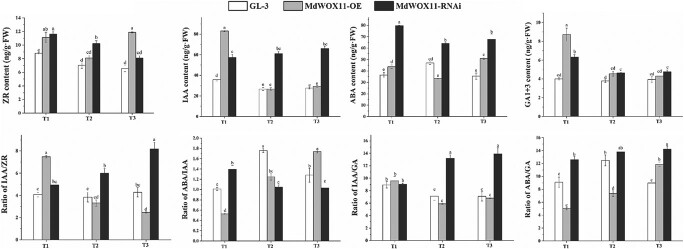
The contents and ratios of ZR, IAA, GA_1 + 3_, ABA, IAA/ZR, IAA/GA, ABA/IAA, and ABA/GA in GL-3 and *MdWOX11* transgenic plants at different stages of AS development. Values represent the mean ± SE of three biological replicates, and letters indicate significant differences between means (*P* < 0.05).

Samples were taken at three stages of AS development in GL-3 and *MdWOX11* transgenic plants, and hormone levels were measured (Figure 4). The trends in hormone content differed between the two types of transgenic plants at different stages. At T1, zeatin riboside (ZR), IAA, GA, and ABA content were lower in GL-3 than in *MdWOX11* transgenic lines, and ZR and ABA content were higher in *MdWOX11-RNAi* than in *MdWOX11-OE* transgenic lines. By contrast, IAA and GA content were lower in *MdWOX11-RNAi* than in *MdWOX11-OE* transgenic lines. At T2 and T3, IAA and GA content were ranked *MdWOX11-RNAi* > *MdWOX11-OE* > GL-3. The ZR, GA, and ABA contents decreased significantly in *MdWOX11* transgenic plants from T1 to T2, and the contents of IAA and ABA rose from T2 to T3. The IAA/ZR, IAA/GA, ABA/IAA, and ABA/GA ratios were also calculated during AS formation. At T1, the IAA/ZR and IAA/GA ratios were lower in *MdWOX11-OE* than in *MdWOX11-RNAi* transgenic lines, and the ABA/IAA and ABA/GA ratios were higher in *MdWOX11-RNAi* than in *MdWOX11-OE* transgenic lines. At T2 and T3, the IAA/ZR, ABA/IAA, and ABA/GA ratios were higher in *MdWOX11-RNAi* than in *MdWOX11-OE* transgenic lines, but the IAA/GA ratio was lower in *MdWOX11-RNAi*. Moreover, the IAA/ZR, ABA/IAA, and ABA/GA ratios increased gradually during AS formation in *MdWOX11-RNAi*, and the IAA/ZR and IAA/GA ratios decreased from T1 to T2 in *MdWOX11-OE* (Figure 4).

### Analysis of RNA-seq data and identification of DEGs

**Figure 5:**
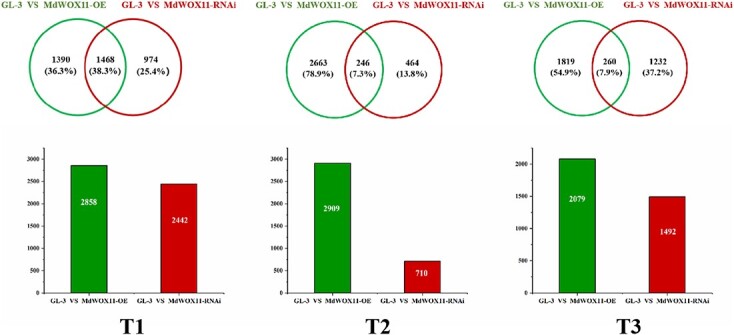
Differentially expressed genes (DEGs) between GL-3 and *MdWOX11* transgenic plants during AS regeneration. GL-3, *MdWOX11-OE*, and *MdWOX11-RNAi* leaves were cultured in shoot induction medium for 0 d (T1), 15 d (T2), and 21 d (T3).

To identify genes specifically associated with AS formation, GL-3 and the transgenic lines were sampled at T1, T2, and T3 for RNA-seq analysis. A Venn diagram was constructed to show differentially expressed genes (DEGs) in *MdWOX11-OE* and *MdWOX11-RNAi* plants relative to GL-3 at three stages of AS development. At T1, there were 2858 and 2442 DEGs between GL-3 and *MdWOX11-OE* and *MdWOX11-RNAi*, respectively; these numbers were 2909 and 710 at T2, and 2079 and 1492 at T3 (Figure 5). In total, there were 3832, 3373, and 3311 DEGs between GL-3 and the transgenic lines at T1, T2, and T3 (8066 DEGs across all AS developmental stages). We performed enrichment analysis of the DEGs, and the results are shown in Supplemental Figure S2. A total of 138 enriched KEGG pathways were identified in the DEGs; these included plant hormone signal transduction, enzyme signal pathways, sugar metabolism–related pathways, and others. GO enrichment analysis of DEGs was also performed, and the results are presented in Supplemental Figure S3.

### Cluster analysis of the expression profiles of hormone-related genes (IAA, CK, GA, and ABA) in *MdWOX11* transgenic plants during AS formation

**Figure 6:**
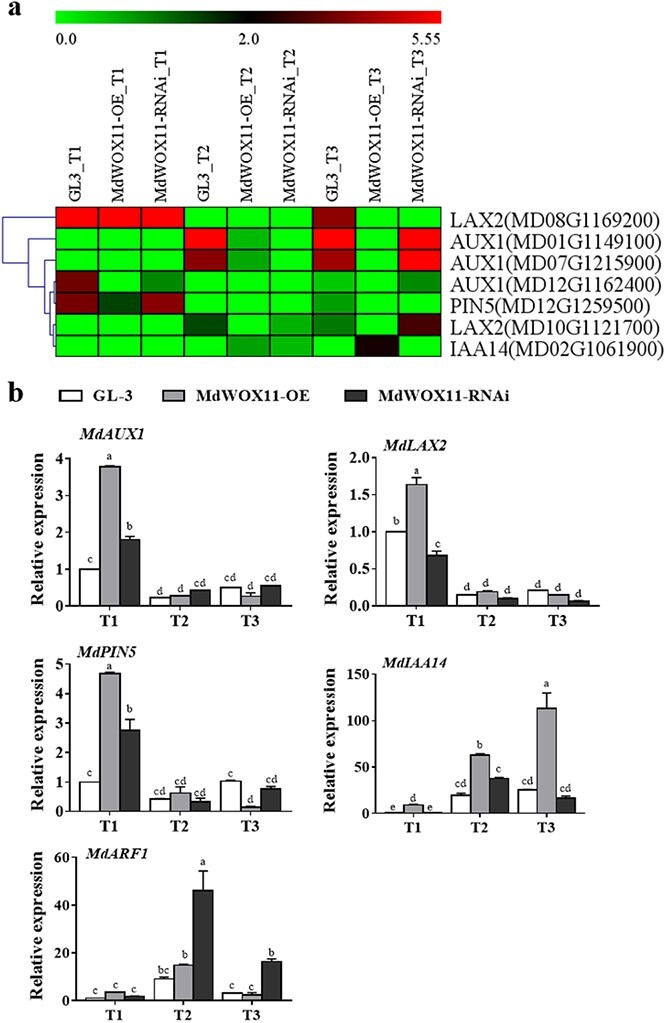
Differential expression of genes related to auxin transport and signal transduction at different stages of AS development in GL-3 and *MdWOX11* transgenic plants. a: Heatmap of log_2_(FPKM) values for auxin-related genes. b: Relative expression of genes related to auxin transport and signal transduction at different stages of AS development in GL-3 and *MdWOX11* transgenic plants. Values represent the mean ± SE of three biological replicates, and letters indicate significant differences between means (*P* < 0.05).

**Figure 7:**
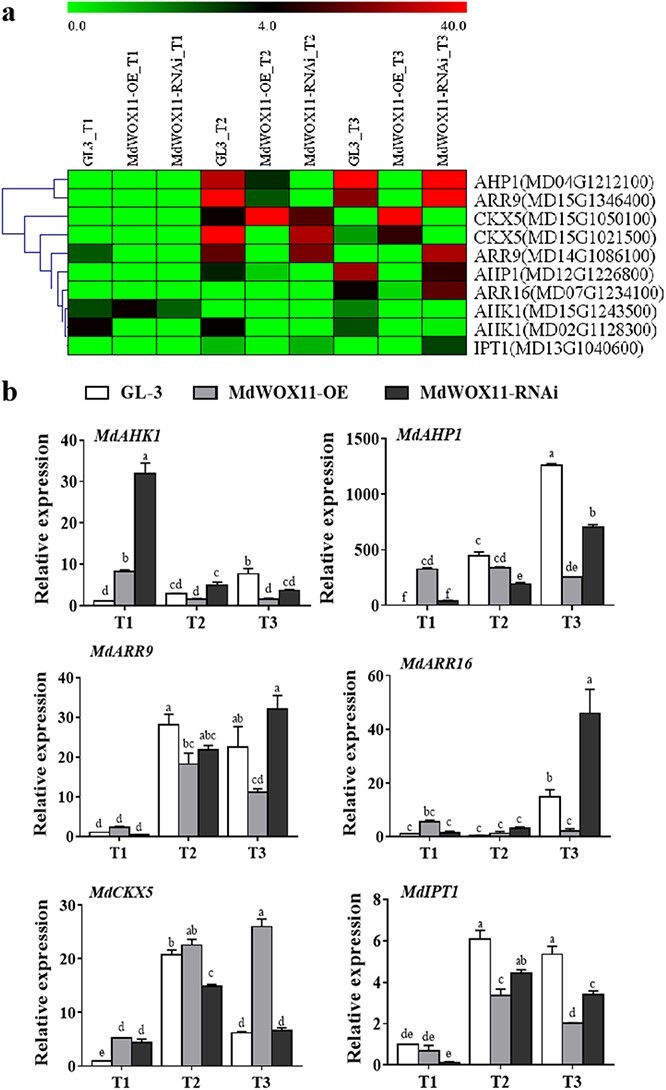
Differential expression of CK-related genes at different stages of AS development in GL-3 and *MdWOX11* transgenic plants. a: Heatmap of log_2_(FPKM) values for CK-related genes. b: Relative expression of CK-related genes at different stages of AS development in GL-3 and *MdWOX11* transgenic plants. Values represent the mean ± SE of three biological replicates, and letters indicate significant differences between means (*P* < 0.05).

**Figure 8:**
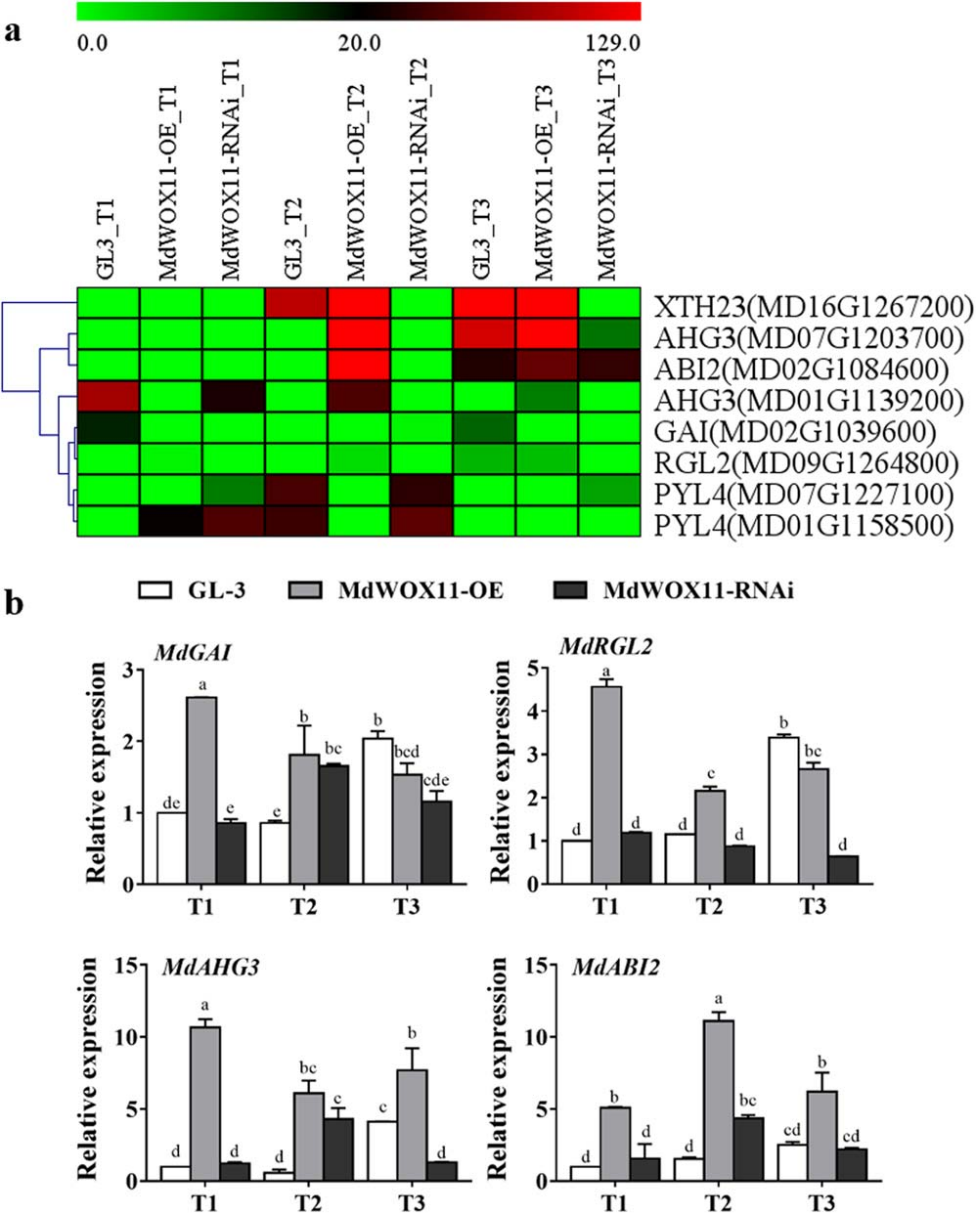
Differential expression of ABA- and GA-related genes at different stages of AS development in GL-3 and *MdWOX11* transgenic plants. a: Heatmap of log_2_(FPKM) values for ABA- and GA-related genes. b: Relative expression of ABA- and GA-related genes at different stages of AS development in GL-3 and *MdWOX11* transgenic plants. Values represent the mean ± SE of three biological replicates, and letters indicate significant differences between means (*P* < 0.05).

Hormone–related genes were analyzed in the *MdWOX11* transgenic plants during AS formation. Expression levels of the IAA transport–related genes *MdAUX1*, *MdLAX2*, and *MdPIN5* were higher at T1 than at T2 and T3, and their expression levels were higher in *MdWOX11-OE* transgenic lines at T1. Expression levels of the IAA signal transduction–related genes *MdIAA14* and *MdARF1* were higher during T2 and T3, and their expression levels were higher in *MdWOX11-OE* transgenic lines than in GL-3 and *MdWOX11-RNAi*. Expression of *MdARF1* was highest in *MdWOX11-RNAi* transgenic lines at T2 and T3, and its expression was more than five times higher in *MdWOX11-RNAi* transgenic lines than in GL-3 plants (Figure 6). Expression levels of the CK signal transduction–related genes *MdAHP1* and *MdARR16* were higher at T3; levels of *MdARR9*, *MdCKX5*, and *MdIPT1* were higher at T2 and T3; and levels of *MdAHP1*, *MdAHK1*, and *MdARR16* rose gradually during AS formation in GL-3. At T2 and T3, expression levels of *MdAHP1* and *MdIPT1* were higher in GL-3 plants than in *MdWOX11-RNAi* and *MdWOX11-OE* transgenic lines, and expression of *MdCKX5* was higher in *MdWOX11-OE* transgenic lines than in *MdWOX11-RNAi* and GL-3 (Figure 7). Expression levels of the GA-related genes *MdGAI* and *MdRGL2* were higher in *MdWOX11-OE* transgenic lines than in GL-3 and *MdWOX11-RNAi* at T1 and T2. At T3, their expression levels were highest in GL-3. Expression levels of the ABA-related genes *MdABG3* and *MdABI2* were higher in *MdWOX11-RNAi* transgenic lines than in GL-3 and *MdWOX11-OE* during AS formation (Figure 8).

### Cluster analysis of the expression profiles of shoot development–related genes in *MdWOX11* transgenic plants during AS formation

**Figure 9:**
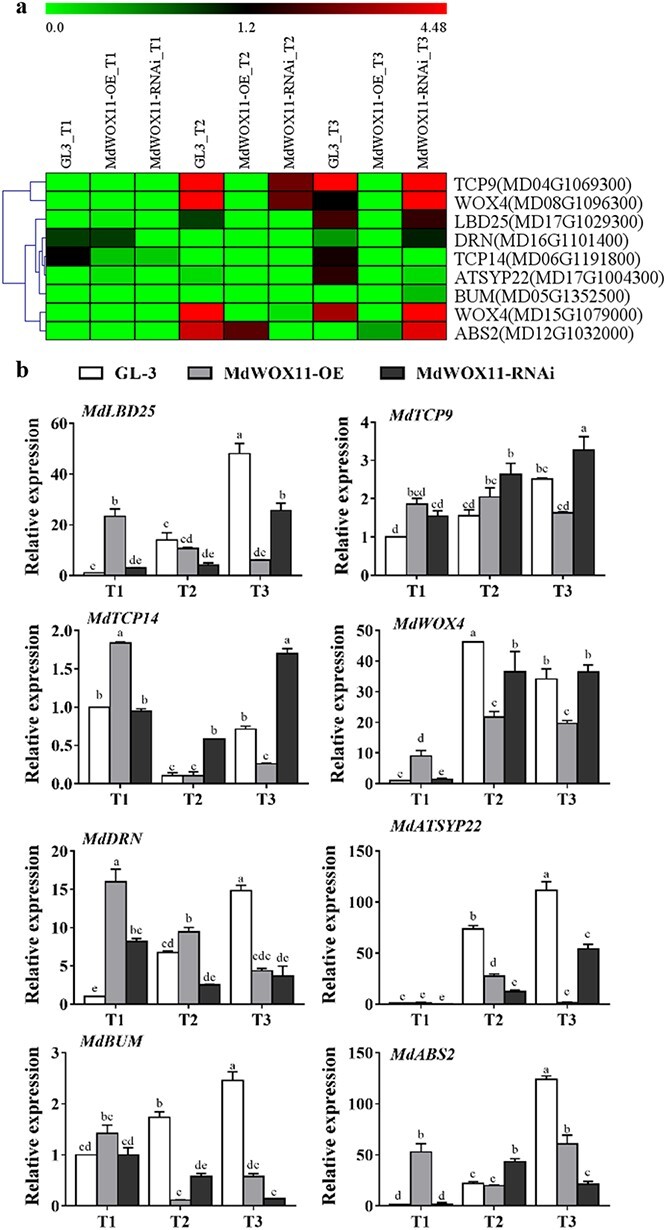
Differential expression of shoot development–related genes at different stages of AS development in GL-3 and *MdWOX11* transgenic plants. a: Heatmap of log_2_(FPKM) values for AS development–related genes. b: Relative expression of shoot development–related genes at different stages of AS development in GL-3 and *MdWOX11* transgenic plants. Values represent the mean ± SE of three biological replicates, and letters indicate significant differences between means (*P* < 0.05).

Shoot development–related genes were analyzed in the *MdWOX11* transgenic plants during AS formation. The expression of most shoot development–related genes increased with AS production. At T1, the expression levels of *MdLBD25*, *MdTCP9*, *MdTCP14*, *MdWOX4*, *MdDRN*, *MdBUM*, and *MdABS2* were higher in GL-3 plants than in *MdWOX11-RNAi* and *MdWOX11-OE* transgenic plants. The expression levels of *MdLBD25*, *MdTCP9*, *MdDRN*, *MdSYP22*, *MdBUM*, and *MdABS2* rose gradually as AS formation progressed in GL-3. At T3 (the AS emergence stage), the expression levels of *MdLBD25*, *MdTCP9*, *MdTCP14*, *MdWOX4*, and *MdSYP22* were lower in *MdWOX11-RNAi* than in GL-3 and *MdWOX11-OE* transgenic lines, whereas the expression levels of *MdDRN*, *MdSYP22*, *MdBUM*, and *MdABS2* were higher in GL-3 than in *MdWOX11-RNAi* and *MdWOX11-OE* (Figure 9).

**Figure 10:**
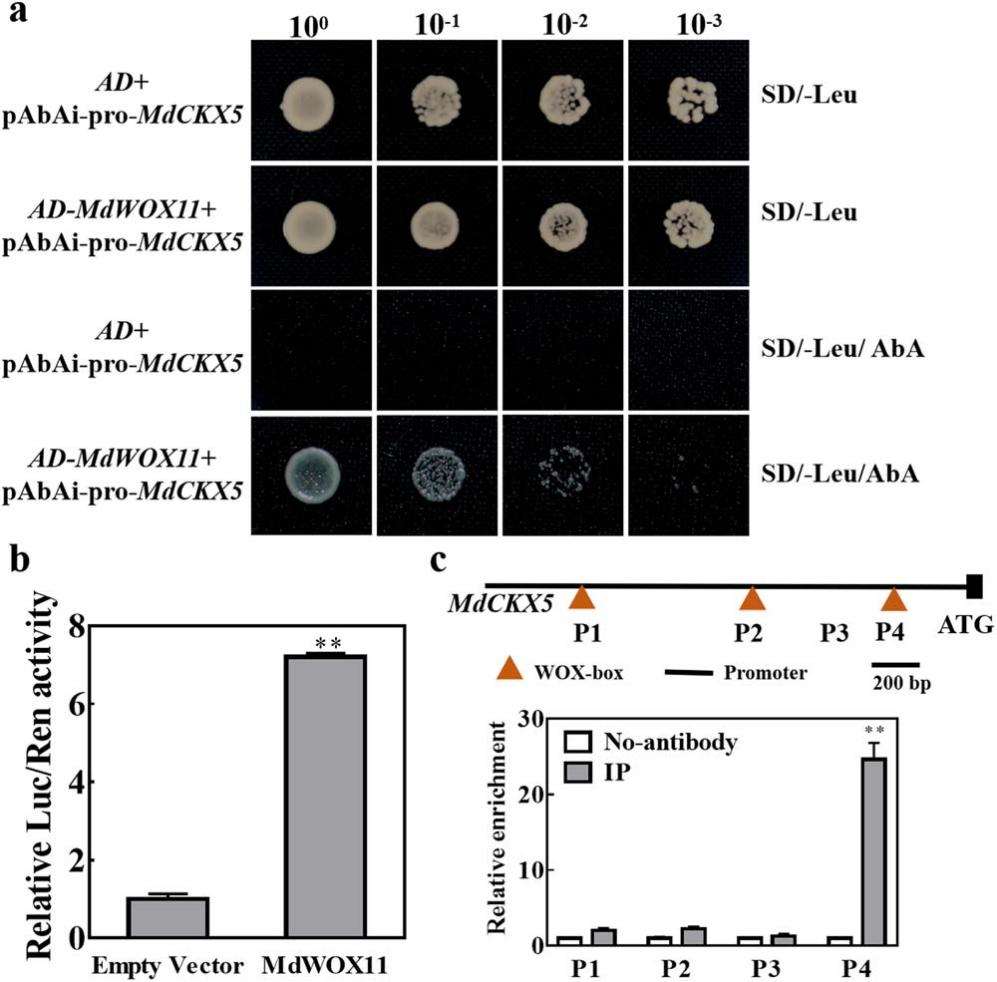
Protein–DNA interactions between MdWOX11 and the *MdCKX5* promoter. a: Yeast one-hybrid assays in which the promoter sequence of *MdCKX5* was cloned and inserted into the pAbAi vector to generate pAbAi-pro-MdCKX5 and the open reading frame of *MdWOX11* was cloned and inserted into the pGADT7 vector to generate AD-*MdWOX11*. AD-Empty indicates the empty pGADT7 vector. b: Dual-luciferase assay results confirmed that MdWOX11 enhances *MdCKX5* promoter activity. Values represent the mean ± SE of three biological replicates, and significant differences were determined using t-tests (^**^P < 0.01). c: ChIP-qPCR analysis of the association of MdWOX11 with promoter fragments of *MdCKX5* using an anti-MdWOX11 antibody; no-antibody indicates the negative control. Values represent the mean ± SE of three biological replicates, and significant differences were determined using the Student’s *t* test (^**^P < 0.01).

To identify genes that act downstream of *MdWOX11*, we analyzed the promoters of the hormone- and shoot development–related genes above. The *MdCKX5* promoter contained the largest number [3] of WOX-binding elements (TTAATGG), and a yeast one-hybrid (Y1H) assay, dual-luciferase assays, and ChIP-qPCR were performed to verify the binding of MdWOX11 to the *MdCKX5* promoter. A Y1H assay showed that MdWOX11 bound to the promoter of *MdCKX5* at a binding site 51–636 bp before the initiation codon (Figure 10a), and this sequence is shown in Figure S4. We next performed transient dual-luciferase assays using MdWOX11 as the effector and the Luc gene under the control of the *MdCKX5* promoter as the reporter to determine whether MdWOX11 regulates *MdCKX5* expression. The Luc/Ren activity was significantly enhanced by MdWOX11 (Figure 10b). *MdWOX11-OE* transgenic leaves during AS development were used for ChIP-qPCR, and the results were consistent with the Y1H results. MdWOX11 bound to the promoter of *MdCKX5* (P4); fragments P1, P2, and P4 contained WOX-box binding elements, whereas P3 did not (Figure 10c).

## Discussion

Adventitious shoots (AS) are buds that grow from stem internodes, roots, leaves, or callus through *in vitro* culture; they differ from terminal, axillary, and accessory buds. AS regeneration is a display of plant totipotency. Some plants may also produce AS near a wound after injury, and the production of AS is a plant response to signals from external stimuli [31]. In this study, injury to the transverse vein served as a signal in leaves. Based on an initial assessment of AS developmental phenotypes through time, we collected samples at three stages of AS development: T1 is the starting point of AS formation, T2 is the stage of callus formation, and T3 is the stage at which green shoot points appeared around the callus. AS formation and regeneration can occur in two ways: the direct proliferation of AS or the indirect proliferation of AS from callus. In this study, most AS formation in apple occurred by indirect proliferation from callus.

Many factors influence the occurrence of AS, and genotype is one of the most important [32]. In this study, we compared the AS regeneration ability of explants and found that SH6 did not produce AS, whereas M26 and MP had a high AS formation ability (Figure 1). Previous research found that the regeneration ability of the leaf blade and petiole was strongly associated with genotype [33]. Hormones are another factor that influences the occurrence of AS. AS regeneration *in vitro* requires the combined action of IAA and CK [34]. CK is commonly used to induce AS formation. The effects of exogenous IAA content are shown in Table S1, and an appropriate CK/IAA ratio was conducive to AS formation.

At the level of gene regulation, most research on *WOX11* function has focused on its regulation of adventitious root formation [35]; little research has been performed on its role in AS formation. Expression analyses of different tissues showed that the expression of *MdWOX11* was lower in leaves than in roots (Figure S1a), and the functions and regulatory mechanisms of *MdWOX11* may therefore be different between roots and leaves. Our expression analysis suggested that *MdWOX11* was a negative regulator of AS formation in apple (Figure 1h). GL-3, as a transgenic apple material, has a strong AS regeneration ability. Analysis of *MdWOX11* transgenic lines showed that *MdWOX11* inhibited AS formation (Figure 2). This is the first study in which *MdWOX11* transgenic lines have been used to investigate the role of *MdWOX11* in the AS formation of apple, and *MdWOX11* clearly plays a key role in increasing the rate of apple leaf regeneration.

We next asked how *MdWOX11* influences hormone levels and hormone-related gene expression to regulate AS formation. We found differences in the endogenous hormone contents of *MdWOX11* transgenic plants at different stages of AS formation (Figure 4). *MdWOX11* transgenesis affected the distribution of endogenous hormones and thereby affected the expression of hormone-related genes. Overexpression of *MdWOX11* increased IAA contents and the expression of IAA transport–related genes at T1 (Figure 4 and 5). Studies on the IAA-responsive promoter *DR5* found that the IAA response was significantly promoted in the callus induction stage [12]. In the early stage of AS regeneration, IAA-related genes such as *AUX/IAA* are upregulated in CIM medium [36]. In this study, the expression levels of the IAA signaling–related genes *MdIAA14* and *ARF1* were upregulated on CIM medium (Figure 6). Furthermore, CK-related genes play an essential role in the regulation of AS formation. Studies have shown that *ARR15* and *ARR16* expression increases during AS regeneration, and an increase in the expression of *ARR15* and *ARR16* was detected at the AS regeneration stage [36]. The expression of CK-regulating genes is also upregulated in SIM culture [37]. In this study, the expression of *ARR9* and *ARR16* was upregulated in SIM medium during the period of AS emergence (Figure 7). The overexpression of the *AHP2* gene in *Arabidopsis* enhanced the effect of CK on roots and hypocotyls [38], suggesting that CK regulates the process of callus conversion to AS formation. Our results showed that *MdAHP1* expression was continuously upregulated in GL-3 and *MdWOX11-RNAi* plants but downregulated in *MdWOX11-OE* transgenic plants (Figure 7). Research has shown that even on medium without CK, plants that overexpressed *IPTs* underwent spontaneous healing through the production of AS on injured tissue [39]. In our study, the expression of *IPT1* genes was higher in GL-3 than in *MdWOX11-OE* transgenic plants (Figure 7). The expression patterns of CK-related genes identified in this study indicated that they play a vital role in callus formation, AS formation, and AS regeneration. The process of AS regeneration is regulated not only by CK but also by hormone interactions. For example, GA is a negative regulatory factor in AS formation [10]. In our study, RNAi of *MdWOX11* influenced GA-related gene expression (Figure 8), providing further evidence that GA is a negative regulator of AS formation.

Among the genes that regulate shoot development, reports have shown that *TCP3* inhibits shoot formation [40], *WUS* and *WOX5* participate in shoot and root development, respectively [41], and *WOX2* plays an important role in shoot apical buds [42]. Overexpression of *WOX4* increased the number of shoot primordia structures induced by wounding and plant hormones [43]. The transcription factor gene *LBD16* is also related to AS regeneration [44]. Here, we documented changes in the expression levels of several transcription factor genes from different families, such as the TCP, LBD, and WOX families. Their expression was lower in *MdWOX11-RNAi* transgenic plants during the period of AS emergence, and these genes may therefore act downstream of *MdWOX11* to regulate AS formation. In addition, *MdDRN*, *MdSYP22*, *MdBUM*, and *MdABS2* are related to shoot formation, and they showed differential expression in the *MdWOX11* transgenic plants, suggesting that they act as positive regulatory factors during AS formation (Figure 9).

**Figure 11:**
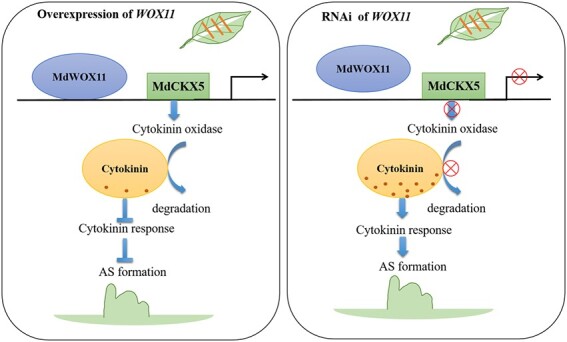
A hypothetical model of MdWOX11 regulating the transcription of *MdCKX5* to mediate AS formation in *MdWOX11* transgenic apple.

Y1H and dual-luciferase assays and ChIP-qPCR showed that *MdCKX5* acts downstream of *MdWOX11* to regulate AS formation (Figure 10). This is the first study to show that WOX11 regulates CK oxidase/dehydrogenase–related genes. Previous studies have shown that WOX11 regulates the expression of CK homeostasis–related genes in rice [45]. Some reports have shown that a decrease in endogenous CK content is directly related to an increase in CKX activity [26]. *Centaurium erythraea* lines that overexpressed *AtCKX1* and *AtCKX2* were characterized by a decline in the average number of spontaneously regenerated shoots [46], and *AtCKX* transgenes affected CK metabolism in transgenic *C. erythraea* plants [47]. OsGRF4 regulated two CK dehydrogenase precursor genes (*CKX5* and *CKX1*), resulting in increased CK levels [48]. Therefore, we speculate that *MdWOX11* inhibited AS formation by activating CK degradation, partly through *MdCKX5* and perhaps through other *MdCKXs*. In *MdWOX11-OE* transgenic plants, MdWOX11 binds to the promoter of *MdCKX5* and induces its expression; *MdCKX5* then promotes the degradation of CK, leaf CK levels decline, and low expression of CK response–related genes inhibits AS formation (Figure 11). In *MdWOX11-RNAi* transgenic plants, transcription of *MdCKX5* is inhibited by low expression of *MdWOX11,* low *MdCKX5* levels limit CK degradation, and high CK content induces the expression of CK response–related genes, leading to AS production (Figure 11). Apple encodes 1500 transcription factors, and bud regeneration is a very complex process. Therefore, a large number of transcription factors and the interactions among physiological signals during callus formation and AS regeneration require further exploration in order to fully describe their underlying molecular mechanisms.

## Conclusions

Genotype and CK/IAA ratio were the key factors that affected AS formation ability in apple leaves. The expression of *MdWOX11* was negatively correlated with AS formation ability, and *MdWOX11* overexpression inhibited AS formation. Y1H and dual-luciferase reporter assays showed that *MdCKX5* acts downstream of *MdWOX11* to control AS formation.

## Methods

### Materials and growth conditions

The tissue-cultured materials originated from buds collected from adult-phase materials in March. Tissue culture cuttings of *M. prunifolia* (MP), *M. zumi* (MZ), M16 × M9 (M26), *Malus honanensis* Rehd. × Ralls Janet (SH6), SZ23, and M9-T337 (T337) were grown at the Northwest Agriculture and Forestry University, Yangling (108°04′ E, 34°16′ N), China under tissue culture conditions and used as cuttings for AS formation. MP, MZ, M26, SH6, SZ23, and T337 were cultured on MS medium with 0.1 mg·L^−1^ indole-3-butyric acid (IBA) and 0.6 mg·L^−1^ 6-benzylaminopurine (6-BA) for 28 days, and fully unfolded young leaves were excised from cuttings for use in regeneration. Cuts were made across the leaf edges and veins to induce AS. A regeneration medium with different proportions of NAA (0.1, 0.5, and 1.0 mg·L^−1^) was designed, and the medium was formulated as MS basal medium with 2.0 mg·L^−1^ TDZ and adjusted to pH 5.8 before sterilization.

### Genetic transformation and growth of transformed apple plants

*35S:MdWOX11-OE* and *35S:MdWOX11-RNAi* transgenic apple plants were produced in the “GL-3” genetic background [49]. The sequence for *MdWOX11* interference is provided in Figure S4. The resulting constructs were transferred into *Agrobacterium tumefaciens* (strain EHA105), and *Agrobacterium*-mediated transformation was used to obtain transgenic apple lines as described previously [50]. Microcuttings from *MdWOX11* transformed lines were harvested for analysis of gene expression. AS induction from leaves of transformed plants was performed as described above. GL-3 was obtained from Prof. Zhihong Zhang (Shenyang Agricultural University, Shenyang, Liaoning). GL-3, *MdWOX11* overexpression (*35S:MdWOX11-OE*), and *MdWOX11* RNA interference (*35S:MdWOX11-RNAi*) transgenic apple plants were cultured on MS medium with 0.2 mg·L^−1^ IBA and 0.3 mg·L^−1^ 6-BA. AS regeneration medium contained 2.0 mg·L^−1^ TDZ and 0.5 mg·L^−1^ NAA. After 3 weeks of dark treatment, the plants were transferred to light culture, and the new medium was replaced once every 4 weeks for 10 weeks of culture.

### Morphological measurements and anatomical observations

A number of morphological parameters were calculated, including the regenerative efficiency, number of shoots per leaf, and the AS increment coefficient. A total of 180 leaves were analyzed, 60 from each group, and leaves were harvested at 0, 7, 15, and 21 d. AS regenerative efficiency (%) was calculated as (number of explants with regenerated AS/total number of explants) × 100%. The average number of AS per explant was calculated as (total number of AS regenerated/number of explants that regenerated AS). The AS increment coefficient was calculated as (total number of AS regenerated/total number of explants inoculated). The collected samples were immediately immersed in liquid nitrogen and stored at −80°C for hormone and RT-qPCR analysis. Anatomical observations were performed using previously described protocols [51–53].

### Hormone extraction and measurement

The 0.1-mg samples for hormone extraction were harvested at 0 d (T1), 15 d (T2), and 21 d (T3) from GL-3 and *MdWOX11* transgenic plants. Hormones were purified and extracted from the harvested samples by a previously described procedure [54]. Three biological replicates were used for each sample and time point. The enzyme-linked immunosorbent assay (ELISA) technique was used to detect and analyze hormones [54].

### RNA sequencing and DEG identification

A total of 27 RNA samples from GL-3 and *MdWOX11* transgenic apple leaves at three AS developmental stages (T1, T2, and T3) were sent to Shanghai Meiji Biomedical Technology for RNA-sequencing and transcriptome assembly using previously described protocols [55]. Fragments per kilobase of transcript per million mapped reads (FPKM) values were used to calculate transcript abundance in GL-3 and *MdWOX11* transgenic plants [56]. A summary of the sequencing statistics for reads and bases obtained in each sample and statistics for the mapping of clean reads from each sample to the reference genome are given in Supplemental Tables S2 and S3. The significance of differentially expressed genes (DEGs) was analyzed using a published method [57]. The biological processes associated with each gene in each sample were evaluated using a Gene Ontology (GO) analysis (http://www.geneontology.org/) [58]. KEGG pathways with corrected *P*-values <0.01 were considered to be significantly enriched (http://www.genome.jp/kegg/).

### Venn diagrams of DEGs and expression profile analyses

DEGs from the 27 leaf libraries were visualized using Venn diagrams [59,60] with VENNTURE software (http://www.irp.nia.nih.gov/branches/lci/nia_bioinformatics_software.html). A cluster analysis was performed with Multiple Experiment Viewer software (MEV4.2) (http://mev.tm4.org/) based on the FPKM values of the genes to generate hierarchical clustering heatmaps.

### Extraction of RNA and synthesis of cDNA

Total RNA was extracted using a CTAB-based method [61], and total RNA integrity was verified with 2% agarose gels. cDNA was synthesized using the Prime Script RT Reagent Kit with gDNA Eraser (TaKaRa Bio, Shiga, Japan).

### RT-qPCR analyses

The expression of *MdWOX11* was analyzed in six apple rootstocks and GL-3. The expressions of genes related to CK, IAA, GA, ABA, and shoot development were analyzed by RT-qPCR in the GL-3 and *MdWOX11* transgenic lines during AS formation. Primers were designed as described previously [62], and gene-specific primers are listed in Supplemental Table S4. RT-qPCR assays were performed as described previously [63], and the apple *ACTIN* gene was used for normalization. Three biological replicates and three technical replicates were tested for each sample. Relative expression of the analyzed genes was calculated by the 2^−ΔΔCt^ method [64].

### Y1H and dual-luciferase reporter assays

The Matchmaker Gold Yeast One-Hybrid System (Clontech, Mountain View, CA, USA) was used for the yeast one-hybrid assays. The CDS of *MdWOX11* was cloned and inserted into the pGADT7 vector (AD-*MdWOX11*), and the promoter sequence of *MdCKX5* (pro-*MdCKX5*) was inserted into the pAbAi vector (*pAbAi-pro-MdCKX5*). *pAbAi-pro-MdCKX5* was transformed into yeast cells, which were plated on SD/−Ura medium supplemented with Aureobasidin A (AbA) to determine the minimal inhibitory concentration of AbA, 500 ng/ml. The AD-*MdWOX11* vector was inserted into yeast cells transformed with pAbAi-pro-*MdCKX5* and selected on SD/−Leu/AbA medium. For dual-luciferase assays, the pro-*MdCKX5* sequence was inserted into the pGreen II 0800-LUC vector, and the CDS of *MdWOX11* was cloned and inserted into the pGreenII 62-SK vector (MdWOX11-SK). The empty pGreenII-62-SK vector was used as the negative control. The constructs were transformed into *Agrobacterium* strain GV3101 and co-transformed into *N. benthamiana* leaves. After 3 d of culture in the dark, LUC and REN activity were quantified by the dual-luciferase reporter assay system (Promega, E1910). At least six biological replications were performed for each co-transformation, and the ratios of LUC to REN were calculated for treatments and controls to assess the binding activity of MdWOX11 to pro-*MdCKX5*.

### ChIP-qPCR

Leaves of *MdWOX11-OE* transgenic plants during AS formation were used to perform ChIP-qPCR. The ChIP assay was performed with anti-MdWOX11 polyclonal antibody produced from rabbit (GenScript, Nanjing, China); no antibody served as the negative control. The primers used for ChIP-qPCR are listed in Supplemental Table S4, and three biological replicates were used for each sample.

### Statistical analysis

The significance of genotype and time point effects were determined using Analysis of Variance (ANOVA), and significant differences between means were determined at the *P* < 0.05 level using SPSS 11.5 software (SPSS, Chicago, IL, USA). Figures were constructed using SigmaPlot 12.0 (Systat Software, Inc.).

## Abbreviations

AS, adventitious shoot; CIM, callus induction medium; SIM, shoot induction medium; SAM, shoot apical meristem; ARF, auxin response factor; RNA-seq, RNA sequencing; TDZ, thidiazuron; NAA, 1-naphthaleneacetic acid; MP, *M. prunifolia*; MZ, *M. zumi*; IAA, auxin; CK, cytokinin; GA, gibberellins; ABA, abscisic acid; ZR, zeatin riboside; WOX5, WUSCHEL-RELATED HOMEOBOX GENE 5; WOX11, WUSCHEL-RELATED HOMEOBOX GENE 11; IBA, indole-3-butyric acid; 6-BA, 6-benzylaminopurine; ELISA, enzyme-linked immunosorbent assay; LBD16, LATERAL ORGAN BOUNDARIES DOMAIN16; DEG, differentially expressed gene;Y1H, yeast one-hybrid; GO, gene ontology; KEGG, Kyoto Encyclopedia of Genes and Genomes; ANOVA, Analysis of Variance.

## Acknowledgments

This work was financially supported by the National Key Research and Development Project (2018YFD1000101, 2019YFD1001803), the Key Research and Development Project in the Shaanxi Province of China (2019TSLNY02-04), Sub-topics of Major Scientific and Technological Project in Shaanxi Province (2020zdzx03-01-04), Tang Scholar by Cyrus Tang Foundation and Northwest Agriculture and Forestry University, the China Apple Research System (CARS-27), and the China Postdoctoral Science Foundation (2020M683584). We would like to thank Prof. Zhihong Zhang (Shenyang Agricultural University, Shenyang, Liaoning) for providing tissue-cultured “GL-3” plants.

## Author contributions

D.Z., J.M., and D.M. designed the research study. D.M. and J.M. performed the research. J.M., D.M., C.N., X.M., K.L., S.C., and X.L. analyzed the data. J.M., D.Z., and M.T. wrote the paper. All authors approved the manuscript.

## Data availability

All relevant data are provided within the paper and its supplementary files.

## Conflict of interest

The authors declare that they have no conflict of interest.

## Supplementary data

Supplementary data is available at *Horticulture Research* online.

## Supplementary Material

Web_Material_uhac080
