# Supplementary material for: Transcriptome analysis reveals the regulatory mechanism by which MdWOX11 suppresses adventitious shoot formation in apple
Source: Hortic Res. 2022 Apr 11;9:uhac080. doi: 10.1093/hr/uhac080 (PMC9160730; doi:10.1093/hr/uhac080)
Supplement: Web_Material_uhac080 [file web_material_uhac080.docx]

Figure S1 Relative expression of *MdWOX11* in GL-3 and *MdWOX11* transgenic plants. a) Relative expression of *MdWOX11* in different tissues, b) Relative expression of *MdWOX11* in GL-3, *MdWOX11-OE* and *MdWOX11-RNAi* transgenic microcuttings, c, d, e) Relative expression of *MdWOX3*, *MdWOX8* and *MdWOX9* in GL-3 and *MdWOX11-RNAi* transgenic microcuttings, f) Relative expression of *MdWOX11* at different stages of AS development in GL-3 and *MdWOX11* transgenic leaves. T1, T2, and T3 were cultured in shoot induction medium for 0 d, 15 d, and 21 d. Values represent the mean ± SE of three biological replicates, and letters indicate significant differences between means (P < 0.05).


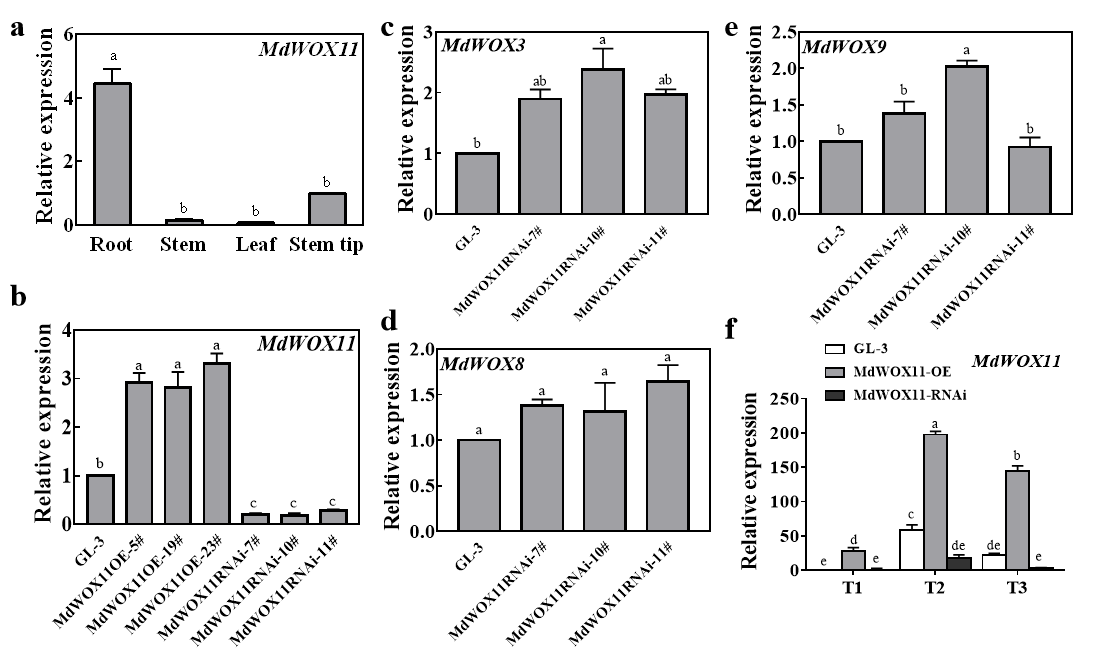


**Figure S2** The KEGG pathway enrichment analysis of the DEGs between the GL-3 and transgenic plants in the formation of adventitious shoots. The vertical axis represents the pathway name, and the horizontal axis represents the Rich factor, which indicates the degree of enrichment. The size of the points indicates the number of genes in the pathway, and the color of the points corresponds to different Qvalue ranges.


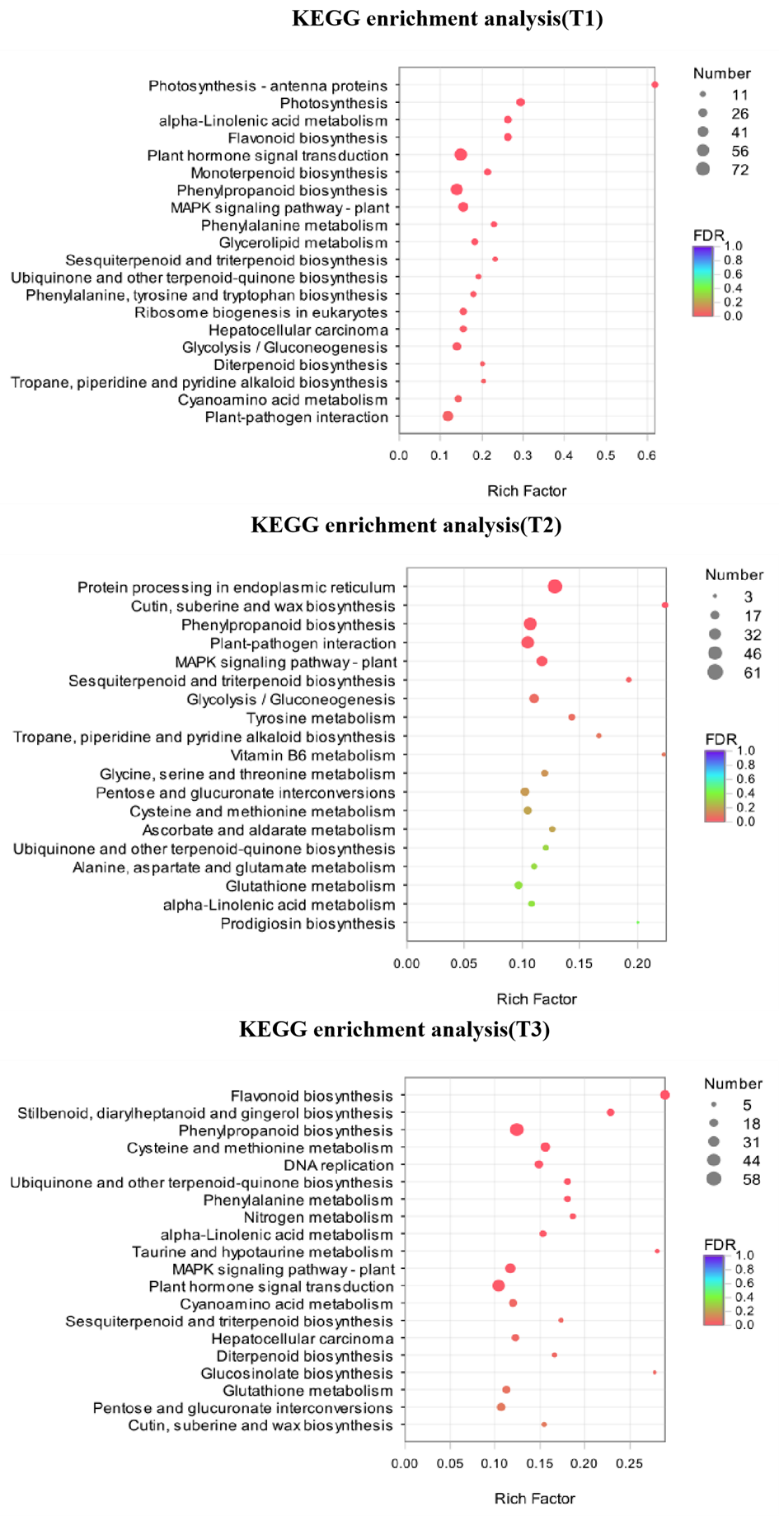


**Figure S3** The GO enrichment analysis of the DEGs between the GL-3 and transgenic plants in the formation of adventitious shoots. The ordinate represents the GO term, and the abscissa represents the significance level of enrichment, which corresponds to the height of the column. The smaller the FDR is, the larger the -log10 (FDR) value is, and the GO term is more significant. The colors represent three major categories: Green represents biological processes (BP), red represents cellular components (CC), and blue represents molecular function (MF).


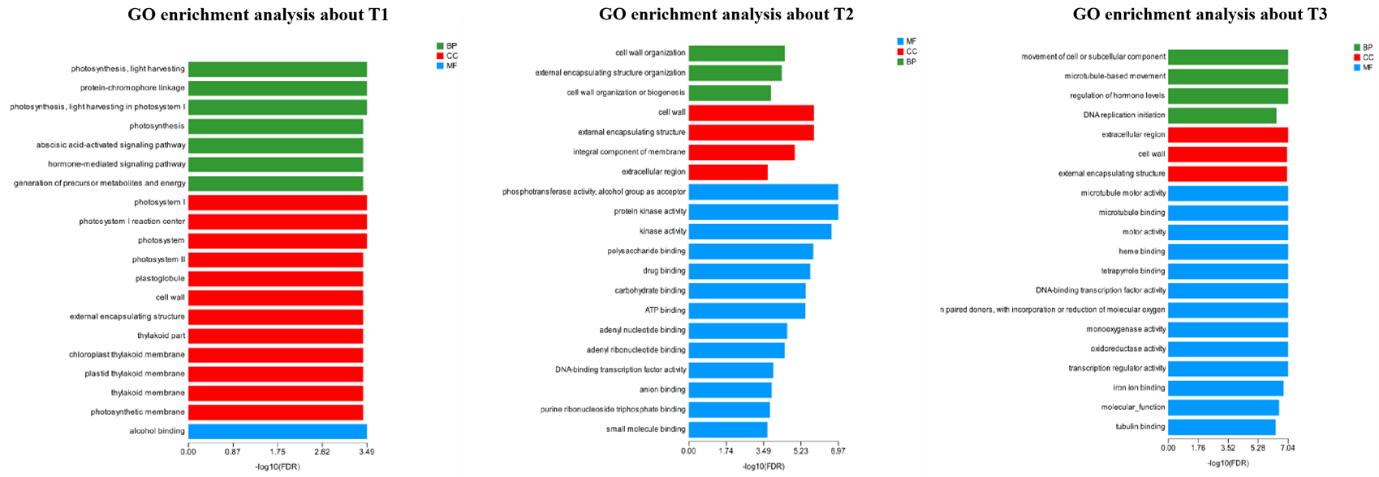


Figure S4. a) The *MdCKX5* promoter sequence was obtained by cloning in ‘GL-3’, red is the WOX-box element. b) The sequence of *MdWOX11* interference.

**a**

**b**

**ATGGAAGATCATCATCAAGGCCAAGACCCTAACGATAGCAGTCCAAGCAACGGCTCGACCAAGAGAAGCCCTGAGCCGGTGAGGTCAAGATGGATACCGAAGCCACAGCAAATCCTAATTCTGGAGTCAATTTTCAACAGTGGAATGGTGAATCCTCCCAAAGAAGAAACTGTGAGAATAAGGAAACTGCTTGAGAAGTTTGGCTCCGTTGGGGATGCCAACGTTTTCTACTGGTTCCAAAACCGACGGTCGCGATCTCGCCGCCGACAACGGCAGTTGCAGGCCAGCCTTGAACAAAGAACCAATTATAATATAAACAATAATCAAATGGCTTCTCTTTCTCAACACCAAGTGGGTGGTGCAATTCAA**

**Table S1**. Comparison of AS formation in seven genotypes of apple materials exposed to different cytokinin to auxin ratios (TDZ/NAA).


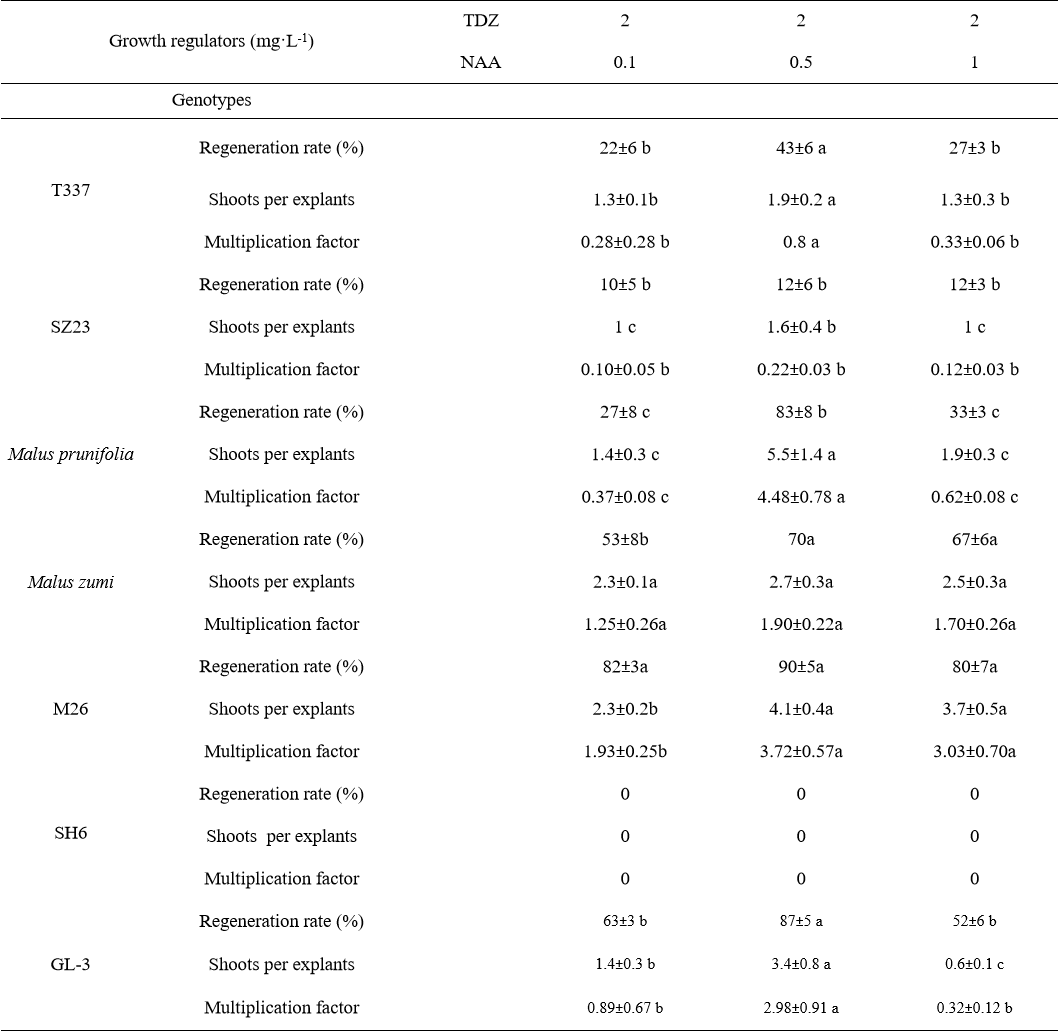


**Table S2**. Summary of the sequencing statistics for the reads and bases obtained in each sample.

| Sample | Raw reads | Raw bases | Clean reads | Clean bases | Error rate  （%） | Q20（%） | Q30（%） | GC content  （%） |
| --- | --- | --- | --- | --- | --- | --- | --- | --- |
| GL3_0d_1 | 58949502 | 8842425300 | 58450180 | 8725836790 | 0.0245 | 98.25 | 94.63 | 47 |
| GL3_0d_2 | 49193408 | 7379011200 | 48776782 | 7273689591 | 0.0245 | 98.24 | 94.63 | 47.21 |
| GL3_0d_3 | 50693226 | 7603983900 | 50257290 | 7505815373 | 0.0245 | 98.23 | 94.58 | 47.08 |
| GL3_15d_1 | 62702950 | 9405442500 | 62168138 | 9292188160 | 0.0235 | 98.63 | 95.64 | 47.34 |
| GL3_15d_2 | 54552900 | 8182935000 | 54092556 | 8081757704 | 0.0235 | 98.63 | 95.64 | 47.17 |
| GL3_15d_3 | 53307602 | 7996140300 | 52863766 | 7893102964 | 0.0235 | 98.65 | 95.7 | 47.18 |
| GL3_21d_1 | 57776016 | 8666402400 | 57280426 | 8548962141 | 0.0236 | 98.6 | 95.58 | 46.95 |
| GL3_21d_2 | 53059928 | 7958989200 | 52613278 | 7856460696 | 0.0235 | 98.65 | 95.71 | 46.93 |
| GL3_21d_3 | 67293128 | 10093969200 | 66708224 | 9966267259 | 0.0236 | 98.58 | 95.5 | 47.12 |
| OE_0d_1 | 57752016 | 8662802400 | 57321886 | 8567354940 | 0.0234 | 98.69 | 95.72 | 47.07 |
| OE_0d_2 | 52555324 | 7883298600 | 52166232 | 7785653032 | 0.0232 | 98.78 | 95.97 | 47.06 |
| OE_0d_3 | 57416210 | 8612431500 | 56985652 | 8507779891 | 0.0234 | 98.7 | 95.74 | 47.05 |
| OE_15d_1 | 56854102 | 8528115300 | 56404002 | 8417349088 | 0.0235 | 98.65 | 95.62 | 46.9 |
| OE_15d_2 | 60724156 | 9108623400 | 60234618 | 8993083100 | 0.0234 | 98.67 | 95.75 | 47.09 |
| OE_15d_3 | 52412536 | 7861880400 | 51988712 | 7762230216 | 0.0234 | 98.67 | 95.74 | 46.91 |
| OE_21d_1 | 52453298 | 7867994700 | 52040248 | 7762337293 | 0.0234 | 98.69 | 95.79 | 46.98 |
| OE_21d_2 | 48889208 | 7333381200 | 48483370 | 7241112969 | 0.0234 | 98.67 | 95.76 | 46.78 |
| OE_21d_3 | 63764636 | 9564695400 | 63249046 | 9448809568 | 0.0236 | 98.62 | 95.55 | 46.81 |
| RNAi_0d_1 | 48271856 | 7240778400 | 47891606 | 7149154881 | 0.0233 | 98.73 | 95.84 | 46.84 |
| RNAi_0d_2 | 50765688 | 7614853200 | 50389336 | 7524530681 | 0.0234 | 98.72 | 95.78 | 46.96 |
| RNAi_0d_3 | 50233410 | 7535011500 | 49860926 | 7449211678 | 0.0233 | 98.72 | 95.81 | 46.95 |
| RNAi_15d_1 | 50940418 | 7641062700 | 50532124 | 7547247549 | 0.0238 | 98.53 | 95.3 | 47.09 |
| RNAi_15d_2 | 53372132 | 8005819800 | 52922654 | 7896006859 | 0.0235 | 98.63 | 95.63 | 47.14 |
| RNAi_15d_3 | 55404056 | 8310608400 | 54954650 | 8196131093 | 0.0235 | 98.66 | 95.71 | 47.23 |
| RNAi_21d_1 | 58581842 | 8787276300 | 58117324 | 8671263795 | 0.0235 | 98.64 | 95.6 | 46.99 |
| RNAi_21d_2 | 49871950 | 7480792500 | 49494546 | 7385668946 | 0.0233 | 98.71 | 95.83 | 46.79 |
| RNAi_21d_3 | 51871066 | 7780659900 | 51435680 | 7664116059 | 0.0235 | 98.65 | 95.68 | 46.95 |

**Table S3.** Summary of the statistics mapped between clean reads of each sample and the reference genome.

| Sample | Total reads | Total mapped | Multiple mapped | Uniquely mapped |
| --- | --- | --- | --- | --- |
| GL3_0d_1 | 58450180 | 54731248（93.64%） | 1775094（3.04%） | 52956154（90.6%） |
| GL3_0d_2 | 48776782 | 45630430（93.55%） | 1398778（2.87%） | 44231652（90.68%） |
| GL3_0d_3 | 50257290 | 47064734（93.65%） | 1511391（3.01%） | 45553343（90.64%） |
| GL3_15d_1 | 62168138 | 58247661（93.69%） | 1801072（2.9%） | 56446589（90.8%） |
| GL3_15d_2 | 60234618 | 56187246（93.28%） | 1797587（2.98%） | 54389659（90.3%） |
| GL3_15d_3 | 52863766 | 49559794（93.75%） | 1556766（2.94%） | 48003028（90.81%） |
| GL3_21d_1 | 54092556 | 50714015（93.75%） | 1552583（2.87%） | 49161432（90.88%） |
| GL3_21d_2 | 52613278 | 49172093（93.46%） | 1496521（2.84%） | 47675572（90.62%） |
| GL3_21d_3 | 66708224 | 62406627（93.55%） | 1952748（2.93%） | 60453879（90.62%） |
| OE_0d_1 | 57321886 | 54034596（94.27%） | 1958195（3.42%） | 52076401（90.85%） |
| OE_0d_2 | 48483370 | 45180974（93.19%） | 1370709（2.83%） | 43810265（90.36%） |
| OE_0d_3 | 56985652 | 53613384（94.08%） | 2162190（3.79%） | 51451194（90.29%） |
| OE_15d_1 | 57280426 | 53466463（93.34%） | 1587717（2.77%） | 51878746（90.57%） |
| OE_15d_2 | 51988712 | 48477256（93.25%） | 1402693（2.7%） | 47074563（90.55%） |
| OE_15d_3 | 56404002 | 52626471（93.3%） | 1563664（2.77%） | 51062807（90.53%） |
| OE_21d_1 | 52040248 | 48550506（93.29%） | 1483104（2.85%） | 47067402（90.44%） |
| OE_21d_2 | 52166232 | 49060948（94.05%） | 1913789（3.67%） | 47147159（90.38%） |
| OE_21d_3 | 63249046 | 58977102（93.25%） | 1826051（2.89%） | 57151051（90.36%） |
| RNAi_0d_1 | 58117324 | 54480731（93.74%） | 1486818（2.56%） | 52993913（91.18%） |
| RNAi_0d_2 | 50389336 | 47464507（94.2%） | 1722537（3.42%） | 45741970（90.78%） |
| RNAi_0d_3 | 49860926 | 46894242（94.05%） | 1700706（3.41%） | 45193536（90.64%） |
| RNAi_15d_1 | 50532124 | 47381421（93.76%） | 1466344（2.9%） | 45915077（90.86%） |
| RNAi_15d_2 | 52922654 | 49620231（93.76%） | 1533930（2.9%） | 48086301（90.86%） |
| RNAi_15d_3 | 54954650 | 51494507（93.7%） | 1724785（3.14%） | 49769722（90.57%） |
| RNAi_21d_1 | 47891606 | 44912162（93.78%） | 1619237（3.38%） | 43292925（90.4%） |
| RNAi_21d_2 | 49494546 | 46247292（93.44%） | 1278454（2.58%） | 44968838（90.86%） |
| RNAi_21d_3 | 51435680 | 48049158（93.42%） | 1426538（2.77%） | 46622620（90.64%） |

**Table S4.** Sequence of primers used for expression analysis, F for the former primer, R for the rear primer, MDP number of gene and length of primers.

| **Gene** | **MDP** | **Sequence 5’-3’** | **length** |
| --- | --- | --- | --- |
| AUX1-F | MD12G1162400 | GAATACAGAACCCGGAAGGGAA | 22 |
| AUX1-R | MD12G1162400 | TCCAAAGAGGAGGAAAGTGCAG | 22 |
| LAX2-F | MD08G1169200 | CCATCTGCGTCTGCTGTGTA | 20 |
| LAX2-R | MD08G1169200 | GGAAGCATGGCTAGGGCATT | 20 |
| IAA14-F | MD02G1061900 | CTCGTGTCTTTGGAGGGTTCA | 21 |
| IAA14-R | MD02G1061900 | ATGATGAGCTGCCAAGTGGT | 20 |
| PIN5-F | MD12G1259500 | GCGGCTATGGTTCCACTCTAC | 21 |
| PIN5-R | MD12G1259500 | ACTGCTCGGGAGTGAAGAC | 19 |
| ARF1-F | MD00G1056300 | GGACTGCTGCTGGAGTGGAA | 20 |
| ARF1-R | MD00G1056300 | CCGTACATCCGCTGCTTTCTAC | 22 |
| AHK1-F | MD15G1243500 | TCACTGCAGAAGATCGTGGAA | 21 |
| AHK1-R | MD15G1243500 | TGGACGGAAACCCCAACTTT | 20 |
| AHP1-F | MD04G1212100 | GAGGGCCTGCATTTCATTCC | 20 |
| AHP1-R | MD04G1212100 | AGCCGCCACAACCTGTCT | 18 |
| ARR9-F | MD15G1346400 | GGGCAGAGGAGTTTCTGTTGA | 21 |
| ARR9-R | MD15G1346400 | GACATTGTTGTCGTCGCTGTT | 21 |
| ARR16-F | MD07G1234100 | TGGGCTTGGGAGATCAACAC | 20 |
| ARR16-R | MD07G1234100 | CATAGCCTGTCATTCCTGGCA | 21 |
| CKX5-F | MD15G1050100 | GCTTACGTGGAGTGCTTCGT | 20 |
| CKX5-R | MD15G1050100 | GTGCCTTGGAGAGGCTTGAG | 20 |
| IPT1-F | MD13G1040600 | CCCCCTAGACGGCGACTT | 18 |
| IPT1-R | MD13G1040600 | TGAAGGAGTTTGACCCGCC | 19 |
| GAI-F | MD02G1039600 | CCTCGAAACCGTCATGGTCA | 20 |
| GAI-R | MD02G1039600 | AACTCGGAGAGGAGGGAGTC | 20 |
| RGL2-F | MD09G1264800 | GAAGTCTCCGGCGCTGTATC | 20 |
| RGL2-R | MD09G1264800 | TGAGTGTGTGGACGAGTTGG | 20 |
| AHG3-F | MD07G1203700 | TATCTAACGACACCGCCTGC | 20 |
| AHG3-R | MD07G1203700 | TCACAGCACAACTCCGACTG | 20 |
| ABI2-F | MD02G1084600 | CGCGTCACCTTTGTTCTTCAA | 21 |
| ABI2-R | MD02G1084600 | GACTTCCCCGATTCACGACT | 20 |
| LBD25-F | MD17G1029300 | TGGCTTCATCGAGTACGAGT | 20 |
| LBD25-R | MD17G1029300 | CTCCGGTGGGAAATAGGGTG | 20 |
| DRN-F | MD17G1110100 | GGGGAGGAGAGAAGAGTGACA | 21 |
| DRN-R | MD17G1110100 | CAGCCGCAGAAACAGAAGTC | 20 |
| TCP9-F | MD04G1069300 | TGGATGTGAGAAAGGACGCC | 20 |
| TCP9-R | MD04G1069300 | GCATTTGCCGGAACGATCAA | 20 |
| TCP14-F | MD06G1191800 | TTAAATCAAATGGGCGGCGG | 20 |
| TCP14-R | MD06G1191800 | CTGACTATGTGGGTGGCTGG | 20 |
| ATSYP22-F | MD14G1229500 | TCAAGGAACAGAAGCCCTGC | 20 |
| ATSYP22-R | MD14G1229500 | ACTTAGGGAAGGGAGTGCCA | 20 |
| WOX4-F | MD15G1079000 | AGAGAAAGTGTAGGAGCTGGGA | 22 |
| WOX4-R | MD15G1079000 | CAGAGTTCTACCTCCCTCAACG | 22 |
| BUM-F | MD15G1006000 | CTTGTGCAGGTTCGTTCTGC | 20 |
| BUM-R | MD15G1006000 | GGCGGTGGATGACTCTCTTT | 20 |
| ABS2-F | MD14G1160900 | CGTGTTTTTCCACGGACTCATTG | 23 |
| ABS2-R | MD14G1160900 | TTCTGATTTCAAACAACGGCCTC | 23 |
| EF-α-F |  | ATTCAAGTATGCCTGGGTGC | 20 |
| EF-α-R |  | CAGTCAGCCTGTGATGTTCC | 20 |
| ACTIN-F |  | TGACCGAATGAGCAAGGAAATTACT | 25 |
| ACTIN-R |  | TACTCAGCTTTGGCAATCCACATC | 24 |
| CKX5-P1-F | MD15G1050100 | TCGTAAAGCAAATAGGAGGACGAA | 24 |
| CKX5-P1-R | MD15G1050100 | TGTAGGTGTTGGTGGTTGGATT | 22 |
| CKX5-P2-F | MD15G1050100 | ACAGGACACTGGACCTATACCT | 22 |
| CKX5-P2-R | MD15G1050100 | GGGATTAGGGTTATTGGGTAGGG | 23 |
| CKX5-P3-F | MD15G1050100 | CCGAATATGGCTCCCCCAAT | 20 |
| CKX5-P3-R | MD15G1050100 | ATGCAATTGAAGCTCCCGTG | 20 |
| CKX5-P4-F | MD15G1050100 | GGCGTTCCAAAAATCTCATTTTGC | 24 |
| CKX5-P4-R | MD15G1050100 | CTTTAGTGGAATTTCAACTTTGCC | 24 |
